# Adapting to the shifting landscape: Implications of climate change for malaria control: A review

**DOI:** 10.1097/MD.0000000000039010

**Published:** 2024-07-19

**Authors:** Emmanuel Ifeanyi Obeagu, Getrude Uzoma Obeagu

**Affiliations:** a Department of Medical Laboratory Science, Kampala International University, Kampala, Uganda; b School of Nursing Science, Kampala International University, Kampala, Uganda.

**Keywords:** climatic change, global public health, malaria, malaria control, vectors

## Abstract

Malaria, a global public health challenge, continues to affect millions of lives, particularly in regions where its transmission is endemic. The interplay between climate change and malaria dynamics has emerged as a critical concern, reshaping the landscape of this vector-borne disease. This review publication, titled “Adapting to the shifting landscape: Implications of climate change for malaria control,” explores the multifaceted relationship between climate change and the control of malaria. The paper begins by dissecting the influence of climate change on malaria dynamics, including alterations in temperature, precipitation, and other climatic factors that impact the habitat and life cycle of malaria vectors. It delves into the evolving ecology and behavior of malaria vectors in response to changing climatic conditions, emphasizing the importance of understanding these adaptations. As a response to this shifting landscape, the review discusses adaptive strategies for malaria control, ranging from vector control measures to the utilization of climate data in early warning systems. Community engagement and education are highlighted as essential components of these strategies, recognizing the vital role of local communities in effective malaria control efforts. The paper also identifies future directions and research needs, underscoring the importance of staying ahead of the evolving climate–malaria relationship. This review underscores the urgency of adapting to the changing landscape of malaria transmission driven by climate change. It emphasizes the significance of proactively addressing climate-related challenges to enhance malaria control and protect the health and well-being of vulnerable populations.

## 1. Introduction

Malaria, a mosquito-borne infectious disease caused by the Plasmodium parasite, has long been a formidable global health challenge, responsible for substantial morbidity and mortality, particularly in regions where it is endemic.^[1,2]^ The fight against malaria has been marked by significant progress, but the emergence of climate change as a pivotal factor is reshaping the landscape of this age-old battle.^[3]^ This paper, titled “Adapting to the Shifting Landscape: Implications of Climate Change for Malaria Control,” embarks on a comprehensive exploration of the intricate and evolving relationship between climate change and the strategies employed in the control of malaria. Malaria, transmitted to humans through the bites of infected female Anopheles mosquitoes, is a disease with multiple dimensions.^[4–7]^ The ecological dynamics of the disease, the behavior of its vectors, and the socioeconomic and public health aspects are all influenced by the ever-changing climate patterns of our planet.^[8]^ As our world experiences shifts in temperature, rainfall, and weather patterns due to global climate change, these shifts, in turn, have profound implications for the distribution, transmission, and control of malaria.^[9]^

In this paper, we navigate through the complex web of interactions between climate change and malaria dynamics. We aim to provide an in-depth understanding of how alterations in temperature and precipitation influence the habitats and life cycles of malaria vectors. We delve into the shifting ecology and behavior of these vectors as they adapt to the changing climatic conditions. The recognition of this adaptation is fundamental to our capacity to anticipate and mitigate the challenges it presents. Our journey continues with an exploration of adaptive strategies for malaria control in the context of climate change. We assess the effectiveness of interventions ranging from insecticide-treated bed nets (ITNs) to indoor residual spraying. Additionally, we highlight the growing importance of climate data in early warning systems for epidemic preparedness and response. Understanding the climate–malaria relationship is fundamental to the development of more targeted and resilient control measures.

While these interventions are pivotal, the human element in malaria control should not be underestimated.^[10]^ We emphasize the significance of community engagement and education in these adaptive strategies, as they empower local communities to take an active role in mitigating the impact of climate change on malaria transmission. This paper seeks to contribute to the collective effort of scientists, healthcare professionals, policymakers, and communities worldwide in adapting to the shifting landscape of malaria. By proactively addressing the implications of climate change on malaria control, we aim to enhance our ability to protect vulnerable populations and achieve a future where the burden of this disease is alleviated.

## 2. Climate change and malaria dynamics

Climate change has a profound impact on the dynamics of malaria, a mosquito-borne infectious disease caused by the Plasmodium parasite.^[11]^ The relationship between climate and malaria is intricate and multifaceted.^[12]^ Malaria transmission is highly sensitive to temperature. Both the development of the Plasmodium parasite within the mosquito vector and the maturation of the mosquito itself are temperature-dependent.^[13]^ Precipitation patterns influence the availability of breeding sites for Anopheles mosquitoes, the primary vectors of malaria.^[14]^ Excessive rainfall can create stagnant water bodies where mosquitoes breed, increasing the risk of transmission.^[15]^ Conversely, extended droughts can reduce breeding sites and decrease transmission.^[16]^ Changes in temperature due to climate change can lead to shifts in the geographic range of malaria. As higher altitudes warm, areas that were once malaria-free may become vulnerable to transmission.^[17]^ This phenomenon has been observed in some regions, including highland areas. Climate change can alter the seasonality of malaria transmission.^[18]^ In some regions, warming temperatures may extend the transmission season, while in others, variations in rainfall can create more irregular patterns of transmission. Extreme weather events, such as heavy rainfall and flooding, can create conditions conducive to rapid increases in mosquito populations, leading to localized outbreaks of malaria.^[19]^ Conversely, droughts can reduce water availability, limiting breeding sites. Changes in climate can also affect the behavior of malaria vectors. For instance, altered temperature and humidity conditions may impact the feeding and resting behavior of mosquitoes, potentially influencing transmission dynamics.^[20]^

Climate change can disrupt communities and affect their ability to implement effective malaria control measures, such as bed net usage and indoor residual spraying.^[21]^ Vulnerable populations may be less equipped to adapt to shifting malaria dynamics.^[22]^ Climate change can influence human migration patterns as people seek more habitable and economically viable areas. This can potentially introduce the parasite to new regions, leading to new transmission dynamics.^[23]^

Understanding the interplay between climate change and malaria dynamics is crucial for adapting control strategies. It highlights the need for proactive measures such as early warning systems, improved access to healthcare, vector control, and community education. Additionally, the development of climate-informed strategies is essential to mitigate the potential expansion and resurgence of malaria in a changing climate.^[24]^ Adaptation and resilience in the face of these shifting dynamics are critical to effectively combat this devastating disease in the years to come.

## 3. Vector ecology and behavior

Vector ecology and behavior play a pivotal role in the transmission of malaria, a mosquito-borne infectious disease caused by the Plasmodium parasite.^[25]^ Anopheles mosquitoes, particularly Anopheles gambiae and Anopheles funestus species, are the primary vectors responsible for transmitting the disease. Understanding the ecology and behavior of these vectors is essential for effective malaria control.^[26]^ Anopheles mosquitoes typically lay their eggs in aquatic habitats, such as stagnant water bodies, puddles, rice fields, and temporary rainwater pools. The type, availability, and location of breeding sites are critical factors in determining the abundance and distribution of malaria vectors.^[26]^ Anopheles mosquitoes are primarily nocturnal and exhibit a preference for feeding on human hosts. This anthropophilic behavior is a significant factor in malaria transmission.

Mosquitoes feed on the blood of an infected individual, acquiring the Plasmodium parasite, which can then be transmitted to other humans during subsequent blood meals.^[26]^ After feeding, Anopheles mosquitoes often rest in dark, sheltered areas to digest their blood meal and avoid environmental threats.^[27]^ Resting behavior is essential for targeting vector control interventions like indoor residual spraying and the use of ITNs.^[28]^ The activity of Anopheles mosquitoes can vary with the seasons and climate.^[26]^ In some regions, transmission may be highly seasonal, while in others, it can be more consistent year-round. Changes in temperature and precipitation can impact mosquito activity and breeding. Temperature affects mosquito development, survival, and the rate at which they transmit malaria. Warmer temperatures can accelerate the development of the Plasmodium parasite within the mosquito, increasing transmission potential.^[29]^ The lifespan of Anopheles mosquitoes can vary, with some species living for several weeks to several months. Longevity impacts their capacity to transmit malaria over time.^[30]^ Not all Anopheles mosquitoes are equally efficient at transmitting malaria. Some species or populations may exhibit higher vector competence, meaning they are more likely to transmit the parasite.^[26]^ An emerging concern is the development of insecticide resistance in Anopheles mosquitoes. Overexposure to insecticides can lead to resistance, reducing the effectiveness of control measures.^[31,32]^

Understanding the ecological and behavioral characteristics of malaria vectors is crucial for the development of targeted and effective control strategies. These strategies may include the use of ITNs, indoor residual spraying, larval source management, and community education. Monitoring changes in vector behavior, insecticide resistance, and climate-related alterations is essential to adapt control efforts and combat malaria effectively.

## 4. Adaptive strategies for malaria control

Adaptive strategies for malaria control are essential in the face of evolving challenges, including changes in the behavior of malaria vectors and the impact of climate change.^[33]^ ITNs have proven highly effective in reducing the transmission of malaria.^[34]^ These nets are treated with insecticides that kill or repel malaria vectors. Adaptive strategies include promoting the use of long-lasting insecticidal nets and monitoring and addressing insecticide resistance in mosquito populations. Indoor residual spraying involves the application of insecticides to the interior walls of houses to kill mosquitoes that come into contact with the treated surfaces.^[35]^ Adaptation involves selecting effective insecticides, addressing resistance, and timing indoor residual spraying to coincide with mosquito activity. Larval Source Management aims to reduce mosquito breeding sites by targeting and treating standing water bodies where mosquito larvae develop.^[36]^ Adaptive strategies include identifying and prioritizing breeding sites, using environmentally friendly larvicides, and conducting community-based source reduction.

Seasonal malaria chemoprevention involves the administration of antimalarial drugs to children in areas with highly seasonal malaria transmission.^[37]^ Adaptation includes adjusting drug distribution timing to align with the malaria transmission season and monitoring for drug resistance. Intermittent Preventive Treatment in Pregnancy provides pregnant women with antimalarial treatment to prevent the adverse effects of malaria on both the mother and the unborn child.^[38]^ Adaptation includes ensuring regular and timely administration during antenatal care visits. Community Health Workers (CHWs) play a vital role in malaria control by providing diagnosis and treatment at the community level.^[39]^ Adaptation involves ongoing training and support for CHWs, as well as community engagement to increase trust in their services. Given the influence of climate on malaria dynamics, climate-informed strategies are essential.^[40]^ These include the use of climate data for early warning systems and the development of adaptive interventions. The development and deployment of novel vector control tools, such as genetically modified mosquitoes or innovative insecticides, can enhance control efforts.^[41]^ Adaptation includes regulatory oversight and community engagement in the use of novel tools. Raising awareness and promoting behavior change within communities to encourage the use of prevention measures, early diagnosis, and prompt treatment.^[42]^ Adaptation involves tailoring education and messaging to local contexts and languages. Regular monitoring and evaluation of control efforts to assess their impact and adapt strategies based on data and evidence.^[43]^ Adaptation includes adjusting interventions based on surveillance data, feedback, and evolving circumstances. Adaptive strategies for malaria control are essential to address the dynamic nature of malaria transmission.^[44]^ By continually assessing and adapting interventions, communities and healthcare systems can enhance their ability to reduce malaria incidence and its impact on public health.

## 5. Community engagement and education for malaria control

Community engagement and education are fundamental components of effective malaria control programs.^[45]^ These strategies empower communities to take an active role in preventing and managing malaria. When community members are informed and engaged, they can contribute to the success of interventions and promote sustainable changes in behavior. Community engagement and education campaigns aim to raise awareness about malaria, its transmission, and its impact on health and well-being.^[46]^ Information about the signs and symptoms of malaria, preventive measures, and available treatment options is crucial for empowering communities to take action. Education initiatives focus on promoting behavior change within communities.^[47]^ This includes encouraging the use of ITNs, seeking early diagnosis and treatment, and reducing mosquito breeding sites. Tailoring messages to the local context and culture is important for achieving behavior change. Engaging communities in decision-making processes related to malaria control fosters a sense of ownership and responsibility.^[48]^ Communities can become partners in designing and implementing interventions. Encouraging communities to take action against malaria empowers them to protect their health and that of their families. Engaged communities are more likely to mobilize resources and support for malaria control efforts.^[49]^ This can include organizing community clean-up activities to eliminate breeding sites or participating in distribution campaigns for bed nets and antimalarial drugs. Community education can help reduce the burden on healthcare systems by encouraging early diagnosis and treatment at the community level.^[50]^ This is particularly important in resource-constrained settings. Training CHWs and traditional healers to recognize and manage malaria cases is part of strengthening healthcare systems. Community engagement and education provide a platform for dispelling myths and misconceptions about malaria and its treatment. This is crucial for building trust and confidence in healthcare services.^[51]^

Effective communication channels, including community meetings, radio broadcasts, text messages, and local leaders, can be used to disseminate information and engage community members.^[52]^ Leveraging existing social networks and traditional communication methods can enhance the reach of educational campaigns. Feedback from community members and ongoing monitoring of interventions help identify challenges and adapt strategies to local contexts.^[53]^ Continuous communication ensures that community concerns and needs are addressed in a timely manner. Community engagement and education contribute to the sustainability of malaria control efforts. Informed and engaged communities are more likely to continue preventive practices and support interventions over the long term.^[54]^ Community engagement and education are integral to the success of malaria control programs.^[55]^ They promote a sense of shared responsibility and empower individuals and communities to take proactive steps to prevent and manage malaria. These strategies are a key part of a comprehensive approach to reducing the burden of this disease.

## 6. Challenges and limitations in adapting malaria control to climate change

Adapting malaria control to the challenges posed by climate change is crucial for the continued success of efforts to combat this disease.^[22]^ Climate change’s impact on malaria is intricate and multifaceted. It involves changes in temperature, precipitation, extreme weather events, and ecological factors. Understanding these complexities is challenging.^[56]^ Accurate climate and health data are essential for monitoring and adapting to climate-related shifts in malaria transmission.^[57]^ Many regions, particularly in low-resource settings, lack sufficient data, and monitoring infrastructure. Implementing climate-informed strategies requires resources, including funding, trained personnel, and access to technology.^[58]^ Some regions with high malaria burdens may have limited resources for such endeavors.

Vulnerable populations, such as those living in poverty, may be disproportionately affected by the impacts of climate change on malaria.^[59]^ These individuals may have less access to healthcare and fewer resources for adaptive measures. Climate change can affect mosquito behavior, distribution, and breeding patterns.^[60]^ In some cases, traditional vector control methods may become less effective, necessitating the development and implementation of new strategies. Climate change can potentially lead to the resurgence of malaria in areas where it was previously under control.^[61]^ This could result in increased morbidity and mortality. While adaptation strategies are being developed and implemented, there may be gaps in how quickly these strategies can be deployed and how effectively they can address the changing dynamics of malaria transmission.^[22]^ Insecticide resistance in mosquito populations is an ongoing concern. Overreliance on insecticides, even in climate-informed strategies, can exacerbate resistance.^[62]^ Malaria control strategies need to be culturally and contextually relevant to be effective. Adapting strategies to local cultures and practices can be challenging.^[63]^ Some adaptive measures may raise ethical concerns. For example, genetic modification of mosquitoes to reduce transmission may face resistance due to concerns about unintended ecological consequences. Building the capacity of healthcare systems to effectively manage and respond to climate-related shifts in malaria transmission is a long-term process that requires substantial investments.^[64]^ The commitment of governments and leadership at both the national and international levels is crucial for prioritizing climate-informed malaria control.^[65]^

Addressing these challenges and limitations requires a concerted effort from the global health community, policymakers, researchers, and local communities.^[66]^ It involves improving data collection and sharing, enhancing healthcare infrastructure, and supporting research into new and more effective control methods. Climate change adaptation for malaria control is an ongoing process, and continued vigilance and collaboration are essential for its success.

## 7. Future directions and research needs of landscape of climate change and malaria

The evolving landscape of climate change and its impact on malaria requires ongoing research and innovation to adapt and enhance control strategies.^[67]^ Future directions and research need in this field are crucial for staying ahead of the challenges posed by changing climate patterns Improve climate modeling and prediction capabilities to provide more accurate and timely information on climate-related malaria risk.^[68]^ Develop climate models that can project malaria transmission patterns with greater precision. Develop and implement advanced early warning systems that use climate data to predict malaria outbreaks.^[69]^ These systems can facilitate proactive control measures and resource allocation. Research and develop malaria control interventions that are specifically designed to be resilient to climate variability.^[22]^ This includes the development of insecticide-resistant mosquito nets and alternative vector control methods. Investigate innovative vector control strategies that can adapt to changing mosquito behavior and breeding patterns.^[70]^ This may include the use of biological control agents or environmentally friendly larvicides. Continue research into genetically modified mosquitoes that are less competent at transmitting malaria.^[71]^ Assess the ecological impacts and safety of releasing such modified mosquitoes. Explore effective methods for community engagement and education in the context of climate change and malaria.^[72]^ Develop culturally sensitive and locally relevant approaches to behavior change. Improve data collection systems for both climate and health. Enhance surveillance and monitoring of malaria cases, mosquito populations, and climate variables, especially in regions with limited resources.^[73]^ Strengthen healthcare systems to be more adaptable to the changing landscape of malaria. This includes training healthcare workers, improving diagnostic capacity, and ensuring a stable supply of antimalarial drugs.^[74]^ Encourage collaboration between climate scientists, epidemiologists, entomologists, and public health experts. Multidisciplinary research can provide a more comprehensive understanding of the complex interactions between climate and malaria.^[75]^ Research and develop malaria vaccines, including those that target specific Plasmodium species or stages of the parasite’s life cycle.^[76]^ Vaccines can be an effective tool in reducing the burden of the disease. Investigate new antimalarial drugs, especially those that are less prone to resistance and can be used in areas where climate change may impact drug efficacy.^[77]^ Integrate climate adaptation measures into broader health and development policies to create a holistic approach that addresses the health and well-being of vulnerable populations.^[78]^ Promote research that engages local communities in data collection and adaptation strategies, ensuring that their perspectives and needs are considered.^[79]^ Examine the ethical implications of various climate-adaptive strategies, such as genetically modified mosquitoes, and involve stakeholders in decision-making processes.^[80]^ Advocate for global health policies and funding mechanisms that support climate-informed malaria control, ensuring that resources are available to address the unique challenges presented by climate change.^[81]^ The evolving landscape of climate change and malaria requires a proactive and multifaceted approach.^[82]^ By prioritizing these research needs and fostering international collaboration, the global health community can develop and implement effective strategies to mitigate the impact of climate change on malaria and protect the health of vulnerable populations.

## 8. Conclusion

In conclusion, as we stand at the intersection of 2 monumental challenges—the relentless advance of climate change and the persistent threat of malaria—our capacity to adapt and innovate will be pivotal in shaping the future of malaria control. The publication “Adapting to the shifting landscape: Implications of climate change for malaria control” has explored the intricate interplay between these forces and has underscored the necessity of proactive, resilient, and climate-informed strategies. Community engagement and education have emerged as indispensable tools, empowering individuals and communities to take ownership of their health and wellbeing. These strategies not only raise awareness but also foster sustainable behavior change and a sense of shared responsibility.

By addressing the future directions and research needs in this dynamic field, we can harness the power of innovation, data, and adaptation to stay ahead of the changing landscape. Together, we can ensure that the relentless march of climate change does not translate into a resurgence of malaria. Our ability to adapt to this shifting landscape will not only protect lives but also represent a triumph of human ingenuity and collaboration in the face of formidable challenges. The path forward is clear: continuous research, community engagement, and climate-informed strategies must be our compass. As we adapt to the shifting landscape of climate change and malaria, we remain steadfast in our commitment to a world where this ancient disease is brought under control, and the health and well-being of vulnerable populations are safeguarded.

## Author contributions

**Conceptualization:** Emmanuel Ifeanyi Obeagu.

**Methodology:** Emmanuel Ifeanyi Obeagu.

**Supervision:** Emmanuel Ifeanyi Obeagu.

**Validation:** Emmanuel Ifeanyi Obeagu.

**Visualization:** Emmanuel Ifeanyi Obeagu.

**Writing – original draft:** Emmanuel Ifeanyi Obeagu, Getrude Uzoma Obeagu.

**Writing – review & editing:** Emmanuel Ifeanyi Obeagu, Getrude Uzoma Obeagu.
